# Calreticulin gene exon 9 frameshift mutations in patients with thrombocytosis

**DOI:** 10.1038/leu.2013.382

**Published:** 2014-01-17

**Authors:** J Chi, K A Nicolaou, V Nicolaidou, L Koumas, A Mitsidou, C Pierides, M Manoloukos, K Barbouti, F Melanthiou, C Prokopiou, G S Vassiliou, P Costeas

**Affiliations:** 1 The Center for the Study of Haematological Malignancies, Nicosia, Cyprus; 2 Molecular Haematology and Immunogenetics Center, The Karaiskakio Foundation, Nicosia, Cyprus; 3 Department of Haematology, Nicosia General Hospital, Nicosia, Cyprus; 4 Department of Haematology, Limassol General Hospital, Limassol, Cyprus; 5 Haematological Cancer Genetics, The Wellcome Trust Sanger Institute, Cambridge, UK

Somatic frameshift mutations in exon 9 of the calreticulin (*CALR*) gene were recently identified in patients with *BCR-ABL*-negative myeloproliferative neoplasms (MPNs), particularly essential thrombocythemia and myelofibrosis.^1, 2^ Calreticulin is a highly conserved endoplasmic reticulum (ER) luminal Ca^2+^-binding chaperone protein with a critical role in the process of glycoprotein folding and a number of other cellular functions both inside and outside the ER,^3, 4^ and has three domains with different structural and functional properties; a globular N domain, a proline-rich P domain and an acidic C domain.^3, 4^ The disrupted C-terminal region contains a KDEL ER-retention sequence multiple sites with high capacity for Ca^2+^ binding and sites for binding to the cell surface and to blood clotting factors.^5, 6^

The specific role of calreticulin in thrombopoiesis is currently unknown and it is thus not clear how *CALR* mutations may drive MPN. However, some of the functions ascribed to calreticulin offer themselves as candidates. For example, as a Ca^2+^-sensor calreticulin regulates Ca^2+^ homeostasis and the ER stress response, which is important for megakaryocyte maturation and platelet formation.^7^ Interactions of the calreticulin N domain with thrombospondin-1 and LRP1 mediate focal adhesion disassembly,^8^ keratinocyte and fibroblast migration^9^ as well as the expression of *TGF*β*3* and collagen.^4^ Collectively, these processes are related to wound healing and fibrosis, a hallmark of MPN pathology. Furthermore, calreticulin is expressed on the surface of human platelets and binds α_2_β_1_ integrin and glycoprotein VI to mediate platelet–collagen interactions.^10^ Finally, Ca^2+^ binding to the C domain of calreticulin regulates protein–protein interactions with other chaperones such as ERp57, which functions as a STAT3 inhibitor, this effect being enhanced by ER luminal complex formation between ERp57 and calreticulin^11^ as are other chaperone functions of ERp57.^12^ Alterations in the Ca^2+^-binding ability of this region could potentially disrupt interactions between the two chaperones and modulate or abrogate the STAT3 inhibitory mechanism. In support of this hypothesis, activation of STAT3 has been shown in a number of essential thrombocythemia patients that lack *JAK2* mutations,^13^ whereas Ba/F3 cells expressing mutant CALR exhibited STAT5 phosphorylation and interleukin-3-independent growth.^2^

The identification of *CALR* exon 9 mutations in a significant proportion of patients with MPN will improve the diagnosis of these diseases, enhance our understanding of their pathogenesis and open new therapeutic avenues for targeted therapy. However, until now the diagnosis of MPNs in the absence of *JAK2* mutations has relied on specific clinicopathological criteria,^14, 15^ and the frequency of *CALR* mutations in patients presenting with persistent thrombocytosis is unknown. To investigate this, we developed a simple and robust assay for the detection of indels in exon 9 of *CALR* and tested 289 samples from patients referred to our laboratory for investigation of persistent thrombocytosis (platelet count >400 000 per μl) over the last 6 years. All samples were analyzed for the presence of JAK2 V617F, MPL codon 515 and *CALR* exon 9 indel mutations.

The presence of JAK2 V617F was detected by an in-house single-tube semi-nested PCR assay with a sensitivity of 0.5% mutant allele burden. In brief, 50 ng of genomic DNA extracted by QIAamp DNA Blood Mini Kit (Qiagen Inc., Valencia, CA, USA) was amplified with HotStarTaq Master Mix (Qiagen) in a 25-μl reaction with the following primers: 7.5 pmol forward outer 5′-ATCTATAGTCATGCTGAAAGTAGGAGAAAG-3′ 0.75 pmol reverse outer 5′-CTGAATAGTCCTACAGTGTTTTCAGTTTCA-3′ 22.5 pmol nested reverse mutant primer 5′-TTACTTACTCTCGTCTCCACATAA-3′. PCR products were separated by electrophoresis on 3% agarose gels. V617F-positive samples produced a 277-bp band in addition to the 367-bp control band. The MPL mutations were detected using the MPL W515L/K Mutant Screen Kit (Qiagen) as per the manufacturer's instructions.

For the detection of *CALR* mutations, 50 ng of genomic DNA was amplified with 7.5 pmol each of forward and reverse primers. Forward primer (*CALR-F*) used was 5′-TAACAAAGGTGAGGCCTGGT-3′ and the reverse primer used was (*CALR-R*) 5′-GCCTCTCTACAGCTCGTCCTT-3′. The reaction was carried out in 1 × HotStarTaq Master Mix (Qiagen) for 40 cycles after 10 min denaturation/activation at 95 °C, and then 95 °C for 30 s, 55 °C for 30 s and 72 °C for 30 s followed by 10 min incubation at 72 °C. Products were separated by electrophoresis on a 3% agarose gel with 0.5 × Tris-borate-EDTA. In addition, PCR products were analyzed by capillary electrophoresis on an ABI3500 genetic analyzer followed by fragment analysis on GeneMapper Software 4.1 (Applied Biosystems, Forest City, CA, USA). For fragment analysis, PCR was carried out with a 6-FAM-labeled forward primer. All samples with an additional peak to the normal were further analyzed by gel purification and Sanger sequencing.

Of 289 samples tested, 189 (65%) carried a JAK2 V617F mutation and 8 (3%) an MPL codon 515 mutation (7 W515L and 1 W515K). Of the remaining 92 samples, 25 were found to carry a *CALR* exon 9 indel mutation (Figure 1a). Patients with JAK2 V617F mutations had, on average, higher hemoglobin concentration compared with patients with thrombocytosis and none of the three mutations studied. Also, patients with either JAK2 V617F or *CALR* exon 9 mutations had significantly higher platelet counts than patients with thrombocytosis and no mutations (Figure 1b).

Each of the 25 *CALR* mutant samples was found to harbor one of seven different indels; all leading to a +1 frameshift of the open reading frame, including two that have not been previously described. The most common mutation, found in 13 out of the 25 cases, was a 52-bp deletion of nt1172 to nt1223 of the *CALR* complementary DNA (cDNA; NM_004343.3). The second most common mutation was a 5-bp insertion (TTGTC) after position nt1127 of the cDNA found in seven cases. Interestingly, these and all other mutations identified reside within a repetitive region containing two simple and three tandem repeats whose location and sequence context suggests that they have a role in the generation of *CALR* mutations by illegitimate local recombination associated with deletions, insertions and inversions (Figure 2a). In particular, the TTGTC, which starts at the end of the second simple repeat, whereas the 52-bp deletion occurs between two GCAGAGG heptanucleotide stretches (nt1092_1098 and nt1143_1150) (Figure 2a). One novel variant involved a complex 13 bp deletion and an inversion insertion of an AGACAA sequence, complementary to part of the deleted 13 bp. Another patient with a 31-bp deletion appeared to also carry a constitutional change of nt1145C>G such that the mutant protein sequence differed from any of those previously described (Figure 2b). Interestingly, the mutation hotspot contains a stretch of 38 nucleotides without a C or a T (such a stretch is expected to occur every 2.7 × 10^11^ nucleotides assuming random distribution of A, C, G, T). As observed by others, all *CALR* exon 9 mutations identified were associated with loss of the C-terminal KDEL moiety and led to the generation of a novel peptide sequence terminating with the same 36 amino acids. Crucially, this alteration transforms the negatively charged glutamic-acid-rich C terminus of calreticulin to a positively charged arginine-rich region (Figure 2b), and this may have a crucial role in mediating the effects of these mutants.

The assay we describe here employs primers situated outside the *CALR* genomic region affected by any of the indel mutations described so far, and this makes it a useful diagnostic tool in the investigation of thrombocytosis or other findings suggestive of MPN. Most mutations were easily detectable by standard PCR and agarose gel electrophoresis because of the formation of a prominent heteroduplex band migrating slower than the wild-type product in the gel. However, in two of the patients carrying a much lower mutation burden than the rest of the patients, the mutations were not detectable on agarose gel electrophoresis. By contrast, all mutations were detected by PCR followed by capillary gel electrophoresis and fragment analysis (Figure 2c). To determine the sensitivity of PCR fragment analysis, we serially diluted five samples carrying different indel mutations with control DNA. These samples had an estimated mutation burden between 40 and 50% based on relative peak areas of the mutant and wild-type PCR products. In all five samples we were consistently able to detect the mutation after a 1:10 dilution, giving this assay a sensitivity to a mutant allele burden of 5% or less (e.g. Figure 2d).

Altogether, JAK2 V617F, *CALR* exon 9 indel and MPL codon W515 mutations were found in 77% of the patients referred to our laboratory for the investigation of persistent thrombocytosis. We presume that the remaining 23% of the patients either had secondary thrombocytosis or a clonal disorder driven by rare unknown mutations. The high incidence of *CALR* indel mutations in patients with persistent thrombocytosis suggests that *CALR* mutational screening should be included in the routine investigation of persistent thrombocytosis, even before strict criteria for the diagnosis of MPN have been established. The method described here, although simple and easy to perform, has the ability to cover the wide range of *CALR* exon 9 mutations and is sensitive enough to detect low mutation burdens. Also, the identification of previously undiscovered *CALR* exon 9 mutation variants, while giving further validity to our assay, suggests that more such variants are likely to be discovered in MPN patients in the future, making the use of such a generic assay more important.

## Figures and Tables

**Figure 1:**
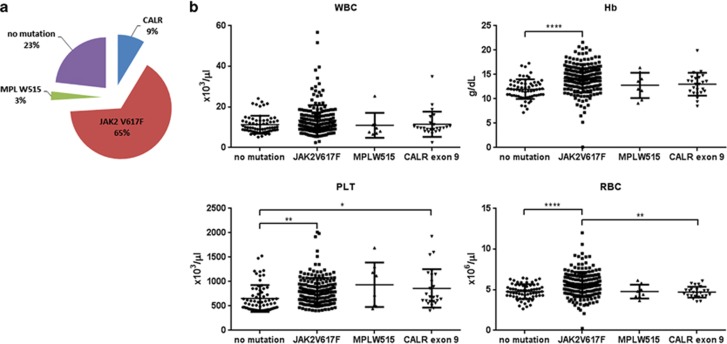
*JAK2*, *MPL* and *CALR* mutation frequency in patients with persistent thrombocytosis and associated hematological parameters. (**a**) Incidence of JAK2 V617F, MPL W515L/K and *CALR* exon 9 mutations in 289 patients investigated for thrombocytosis. (**b**) White blood cell (WBC), hemoglobin (Hb), platelet (PLT) and red blood cell (RBC) counts in the four patient groups studied (*P<0.05, **P<0.01, ****P<0.0001 by one-way analysis of variance with Tukey's multiple testing correction).

**Figure 2:**
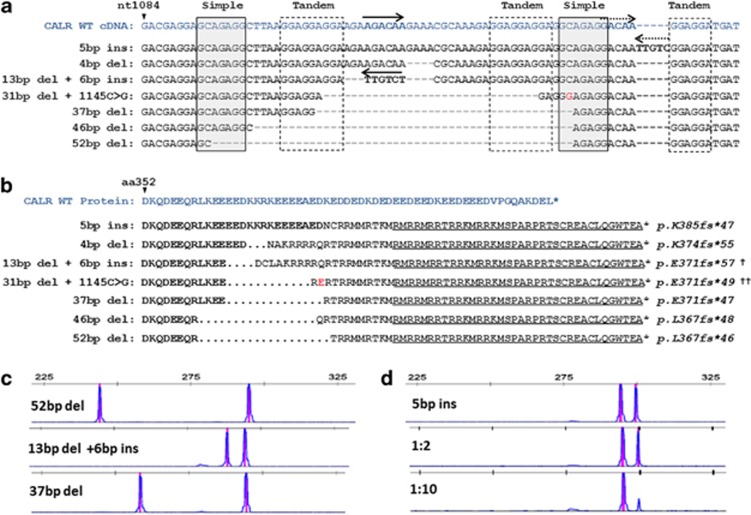
Characterization of *CALR* exon 9 mutations in patients with thrombocytosis. (**a**) Comparative sequences of the seven different types of *CALR* mutations found in this study against the wild-type *CALR* exon 9 cDNA. Repeat elements are highlighted by gray solid lined (simple) or dashed lined (tandem) boxes and inverted sequences are depicted by reverse arrows. Presumed genomic regions from which these inversions may have derived are depicted by matching solid/dashed forward arrows. A polymorphism (G) is depicted in red and this was also found in normal DNA from the same individual. (**b**) Predicted C-terminal amino-acid sequences resulting from the frameshift indel mutations compared with the wild-type calreticulin protein sequence. The common novel peptide sequence shared by all mutations described to date is underlined. The amino-acid variant resulting from the polymorphism above is shown in red (**e**). (**c**) Results of PCR fragment analysis of *CALR* exon 9 in three patients with different indel mutations. (**d**) Example of fragment analysis from a patient with a 5-bp insertion diluted into normal control DNA shows that the mutation is readily detectable after a 1:10 dilution. ^†^Novel mutation variant, ^††^Novel mutant amino-acid sequence.
